# Changes in Cortical Activity in Stroke Survivors Undergoing Botulinum Neurotoxin Therapy for Treatment of Focal Spasticity

**DOI:** 10.3389/fresc.2021.735819

**Published:** 2021-12-16

**Authors:** Kaleb Vinehout, Kelsey Tynes, Miguel R. Sotelo, Allison S. Hyngstrom, John R. McGuire, Brian D. Schmit

**Affiliations:** ^1^Department of Biomedical Engineering, Marquette University and the Medical College of Wisconsin, Milwaukee, WI, United States; ^2^Department of Physical Therapy, Marquette University, Milwaukee, WI, United States; ^3^Department of Physical Medicine and Rehabilitation, Medical College of Wisconsin, Milwaukee, WI, United States

**Keywords:** stroke, rehabilitation, BoNT-A, MRI, activation, connectivity

## Abstract

**Background:** Botulinum NeuroToxin-A (BoNT-A) relieves muscle spasticity and increases range of motion necessary for stroke rehabilitation. Determining the effects of BoNT-A therapy on brain neuroplasticity could help physicians customize its use and predict its outcome.

**Objective:** The purpose of this study was to investigate the effects of Botulinum Toxin-A therapy for treatment of focal spasticity on brain activation and functional connectivity.

**Design:** We used functional Magnetic Resonance Imaging (fMRI) to track changes in blood oxygen-level dependent (BOLD) activation and functional connectivity associated with BoNT-A therapy in nine chronic stroke participants, and eight age-matched controls. Scans were acquired before BoNT-A injections (W0) and 6 weeks after the injections (W6). The task fMRI scan consisted of a block design of alternating mass finger flexion and extension. The voxel-level changes in BOLD activation, and pairwise changes in functional connectivity were analyzed for BoNT-A treatment (stroke W0 vs. W6).

**Results:** BoNT-A injection therapy resulted in significant increases in brain activation in the contralesional premotor cortex, cingulate gyrus, thalamus, superior cerebellum, and in the ipsilesional sensory integration area. Lastly, cerebellar connectivity correlated with the Fugl-Meyer assessment of motor impairment before injection, while premotor connectivity correlated with the Fugl-Meyer score after injection.

**Conclusion:** BoNT-A therapy for treatment of focal spasticity resulted in increased brain activation in areas associated with motor control, and cerebellar connectivity correlated with motor impairment before injection. These results suggest that neuroplastic effects might take place in response to improvements in focal spasticity.

## Introduction

Spasticity occurs in up to 40% of stroke survivors and is associated with functional loss, based on correlations with Barthel scores [see (1, 2) for review]. Botulinum Neurotoxin type A (BoNT-A), which acts by blocking the release of acetylcholine at the neuromuscular junction, temporarily relieves lower limb (3), and upper limb spasticity in patients with stroke (4–6); however, improvements in arm and hand function are not consistently observed across individuals (4, 7). Care and comfort of the hand are consistently improved with BoNT-A injections (8, 9), despite the observation that broader function of the hand is not reliably achieved (9, 10). When outcomes of BoNT-A treatments are considered in terms of passive and active function, passive functional goals are more often met compared to placebo controls, while no differences in active functional goals are observed (11). There is, however, some promise of BoNT-A for targeted improvements in function. A recent titration study demonstrated that increasing the dose of BoNT-A improves the attainment of individual goals (12). With improved goal attainment after BoNT-A treatment, it is possible that there are underlying neuroplastic effects that contribute to functional improvement. Thus, the purpose of the current study was to determine whether there are changes in brain activation or connectivity associated with hand function after BoNT-A that could serve as the basis for restoration of functional movement.

Investigations of brain activation following BoNT-A therapy have produced varying results. In contrast to dominant unilateral activation of motor areas during hand movements in controls, stroke survivors have extensive bilateral activation of primary sensorimotor, premotor and supplementary motor areas during movement of the affected hand (13–15). Changes in brain activity patterns following BoNT-A injections have shown variable results. Some reports indicate a reduction in the bilateral volume of activation in primary motor areas after BoNT-A injections during active (16–18) and imagined (19) movements, demonstrating a localizing and lateralization effect. Other studies have shown increases in activity in similar motor areas after passive wrist movement (20) and arm cycling (21) following BoNT-A therapy. In addition to changes in brain activity patterns after stroke, there are significant alterations in functional connectivity following stroke, including decreases in interhemispheric connections to somatomotor areas and increases in intrahemispheric connections (22–26); however, changes in connectivity with BoNT-A treatments have not been considered, to date.

In this study, we used functional magnetic resonance imaging (fMRI) to investigate the underlying changes in brain activation and connectivity following BoNT-A therapy to relieve upper limb spasticity after stroke. We examined changes in both brain activity and functional connectivity in participants undergoing BoNT-A therapy. To include stroke participants with severe limb spasticity, we developed a wrist-hand device that would allow active finger flexion while passively assisting the fingers to full extension. Note that Bergfeldt et al. (16) and Manganotti et al. (17) also used devices during fMRI measurements to minimize flexor synergies and large synkinetic movements; our device was unique in that it allowed full range-of-motion of finger flexion for stroke participants with mild-to-severe spasticity during fMRI scanning. Our analyses consisted of voxel-based activity measurements and region of interest (ROI) functional connectivity analyses of fMRI data. We hypothesized that BoNT-A's peripheral effects on the affected limb would increase brain activity in higher order motor control centers such as the premotor area and improve global connectivity between motor control centers.

## Methods

In this functional MRI study, we obtained blood oxygen level dependent (BOLD) images from a convenience sample of stroke participants undergoing botulinum toxin therapy for arm spasticity. The BoNT-A treatment was part of prescribed clinical care and was not modified for this study. We measured BOLD activation at the time of injection (W0) and 6 weeks (W6) later, at the peak effect of the botulinum toxin on alleviating arm spasticity. We performed voxel-level whole-brain activation and independent component analyses to identify changes in functional activation and connectivity associated with motor recovery due to botulinum toxin therapy. Nine people with chronic stroke were enrolled in the study (5 female; aged 58.2 ± 3.8, range 42–77). Stroke inclusion criteria included: undergoing BoNT-A therapy as part of clinical care; stroke onset more than 6 months prior to the study; wrist/finger impairment as determined by physical examination; no contraindication to MRI. All participants suffered from upper extremity spasticity following stroke and had previously undergone at least one session of BoNT-A treatment. Eight age-matched controls (3 female; aged 56.4 ± 2.2, range 47–70) were enrolled. Control inclusion criteria included: no known neurological or muscular disease and no MRI contraindication. All procedures were approved by the Institutional Review Board (IRB) of the Medical College of Wisconsin (MCW). All participants gave written informed consent to take part in this study and all procedures were conducted in accordance with the Declaration of the World Medical Association.

### Study Set-Up

This study consisted of two test sessions scheduled 6 weeks apart for both control and stroke participants. For participants receiving BoNT-A therapy, each session included an MRI scan and a clinical assessment. At least 3 months had passed since the patients' last BoNT-A injection before being enrolled in this study. The first session was conducted 1–4 days before participants received their BoNT-A injection (W0), and the protocol was repeated 6 weeks post-injection in the second session (W6). The control group participated only in the imaging portion of the procedures, with the exception of participants C1 and C5, who did not attend the second session. These controls were used as a comparison to stroke participants. All data were processed with the same analysis, except lesions were identified in stroke participants to aid in registration.

### BoNT-A Administration and Clinical Data

Each participant had been treated with BoNT-A 3–4 months prior to the study period as part of their usual standard of care (Table 1). Each participant was prescribed physical therapy following BoNT-A injections; however, only two participants underwent therapy (4 sessions between W0 and W6 in each case: see Table 2). The dosage and muscles injected (Table 2) were determined by the severity of spasticity and the individual's goals for the treatment. EMG guidance was used for all injections. The dose of BoNT-A and muscle injected was not adjusted for study purposes. Summary information of the number of BoNT-A injections, muscles injected, and physical therapy sessions are shown in Table 2.

**Table 1:** Participant demographics and clinical characteristics.

**Participant**	**Sex**	**Age**	**Stroke**	**Lesion**	**Time post**	**MAS**	**FM Pre/Post**	**Nth**	**Physical**
		**(Years)**	**type**	**location**	**stroke (Years)**	**finger/wrist**	**injection**	**injection**	**therapy**
BTX 1	F	48	Isch	R MCA-UD	4.6	3/3	23/26	6	Prescribed
BTX 2	M	58	Hem	L Ip-BG	4.4	2/2	26/27	13	Prescribed
BTX 3	F	42	Hem	L Ip-BG	3.9	3/4	19/22	12	Prescribed
BTX 4	M	77	Isch	L MCA-LD, UD, Le	1.4	3/3	20/22	2	Prescribed
BTX 5	F	67	Isch	R MCA-LD, UD	1.8	3/2	9/9	N/A	N/A
BTX 6	F	60	Isch	R MCA-LD, UD, Le	11.9	1/1	23/25	40	Prescribed
BTX 7	M	69	Isch	L Pons	1.1	2/1	63/63	2	Not Prescribed
BTX 8	F	48	Isch	R MCA-LD, UD	8.9	4/4	44/47	27	Home Exercises
BTX 9	M	55	Isch	R MCA-LD, UD, Le	5.1	4/2	35/40	17	Home Exercises

*MCA, middle cerebral artery; LD, Lower Division; UD, Upper Division; Le, Lenticulostriate; Ip, Intraparenchymal; BG, Basal Ganglia; R, Right; L, Left; Isch, Ischemic; Hem, Hemorrhagic; Note: information regarding the number of injections and details of physical therapy for participant number 5 was not available due to transfer between centers*.

**Table 2:** Therapy dosage.

**Name**	**BoNT**	**DOSE**	**#**	**MUSCLES**
	**#**	**Units**	**THERAPY**	
BX1	B6	450	4	PMj, PMn, LD, TR, BRA, BRD, ECR, FCR, FDP, FPL
BX2	B11	350	0	PMj, BRA, BRD, PT, FCR, FCU, FDS, FDP, FPL
BX3	B12	300	0	BIC, BRD, PT, FCR, FCU, FDS, FDP
BX4	D2	550	4	PMj, LD, PT, FDS, FDP
BX5	B4	200	0	PMj, BIC, FDS
BX6	B40	200	0	LS, LD, PMn, PT, ECR, FPB, Lu
BX7	X2	125	0	LD, BRA, BRD
BX8	B27	325	0	LD, BRA, BRD, FCR, FCU, FDS, FDP, FPL, FPB, Lu
BX9	B17	325	0	PMj, BRA, BRD, PT, ECR, ECU, FDS, FDP, FPB, Lu

*Table listing the BoNT dosage and physical therapy sessions for each stroke participant*.

*#Therapy, therapy sessions between BoNT-A treatments; BoNT B, Botox (OnabotulinumtoxinA); D, Dysport (AbobotulinumtoxinA); X, Xeomin (IncobotulinumtoxinA); PMj, Pect Major; PMn, Pect Minor; LD, Latissimus Dorsi; LS, Levator scap; TR, Tricaps; BRA, Brachialis; BRD, Brachioradialis; PT, Pron Teres; Lu-Lumbricals; FCU, Flexor Carpi Ulnaris; FPB, Flexor Pollicis Brevis; BIC, Biceps Brachii; FDS, Flexor Digitorum Superficialis; FPL, Flexor Pollicis Longus; FCR, Flexor Carpi Radialis; ECR, Extensor Carpi Radialis*.

**Figure 1:**
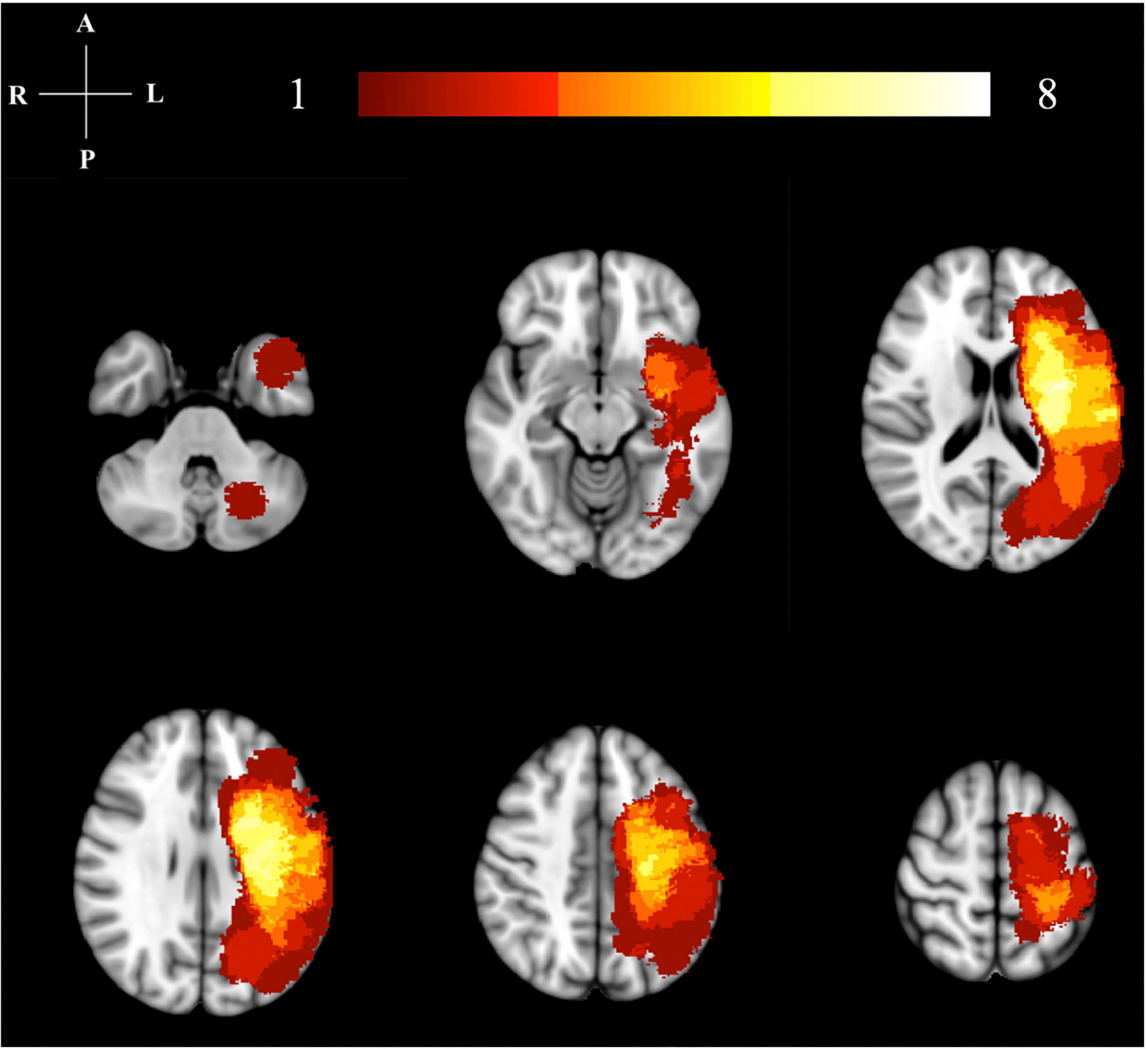
Lesion location map. This image illustrates the lesion distribution for our study. The color bar and numbers represent the number of subjects with a lesion in that area. The highest lesion distribution in shown in bright colors (yellow/white). All right hemisphere lesions have been flipped to the left hemisphere for analysis.

All stroke participants' paretic arm motor impairment was assessed using the Fugl-Meyer Assessment (FMA) (27) at time-points W0 and W6. The wrist and finger flexor spastic hypertonia and increased muscle tone were assessed using the Modified Ashworth Scale (MAS) (28) at W0. The MAS has moderate test-retest reliability (29) very good interrater reliability (30), and convergent validity with the FMA, EMG response to a ramp stretch, and the pendulum test (31). The FMA has high test-retest and interrater reliability in people with stroke (32–34). The W0 and W6 FMA scores were checked for non-normality using the Anderson-Darling test and compared for significant changes with a paired *t*-test. The MAS and FMA measurements were compared with activation volume, activation intensity, and functional connectivity measurements. Comparisons with the FMA scores used a Pearson correlation, while those with the MAS used a Spearman correlation. A Pearson correlation was used for the FMA based on the assumption that the FMA data is measured on an interval scale. In contrast, the MAS was assumed to be an ordinal variable and thus, the Spearman correlation was used. The FMA was collected at both timepoints due to its greater clinical relevance and psychometric properties compared to the MAS (35). Characteristics of the participants in the stroke group are described in Table 1, with further details regarding size and location of the lesions illustrated in Figure 1.

### Imaging Data Acquisition

All images were collected using a 3.0T GE Discovery MR750 scanner equipped with a 32-channel head receive coil (MR Instruments, Inc.; Distributed by GE Healthcare; frequency: 127.73 MHz; field: 3T). Anatomical 3D images were collected using the following fast spoiled gradient echo planar imaging (FSPRG-EPI) protocol: Echo Time (TE) = 3.2 ms, Repetition Time (TR) = 8.16 ms, Field of View (FOV) = 240 mm, and 156 × 1 mm slices. Two 6-min trials were conducted for the fMRI, using a GE's gradient echo planar imaging (GRE-EPI) protocol with the following parameters: TE = 25 ms, TR = 2,000 ms, FOV = 224 mm, Matrix: 64 × 64 mm, and 41 × 3.5 mm sagittal slices.

**Figure 2:**
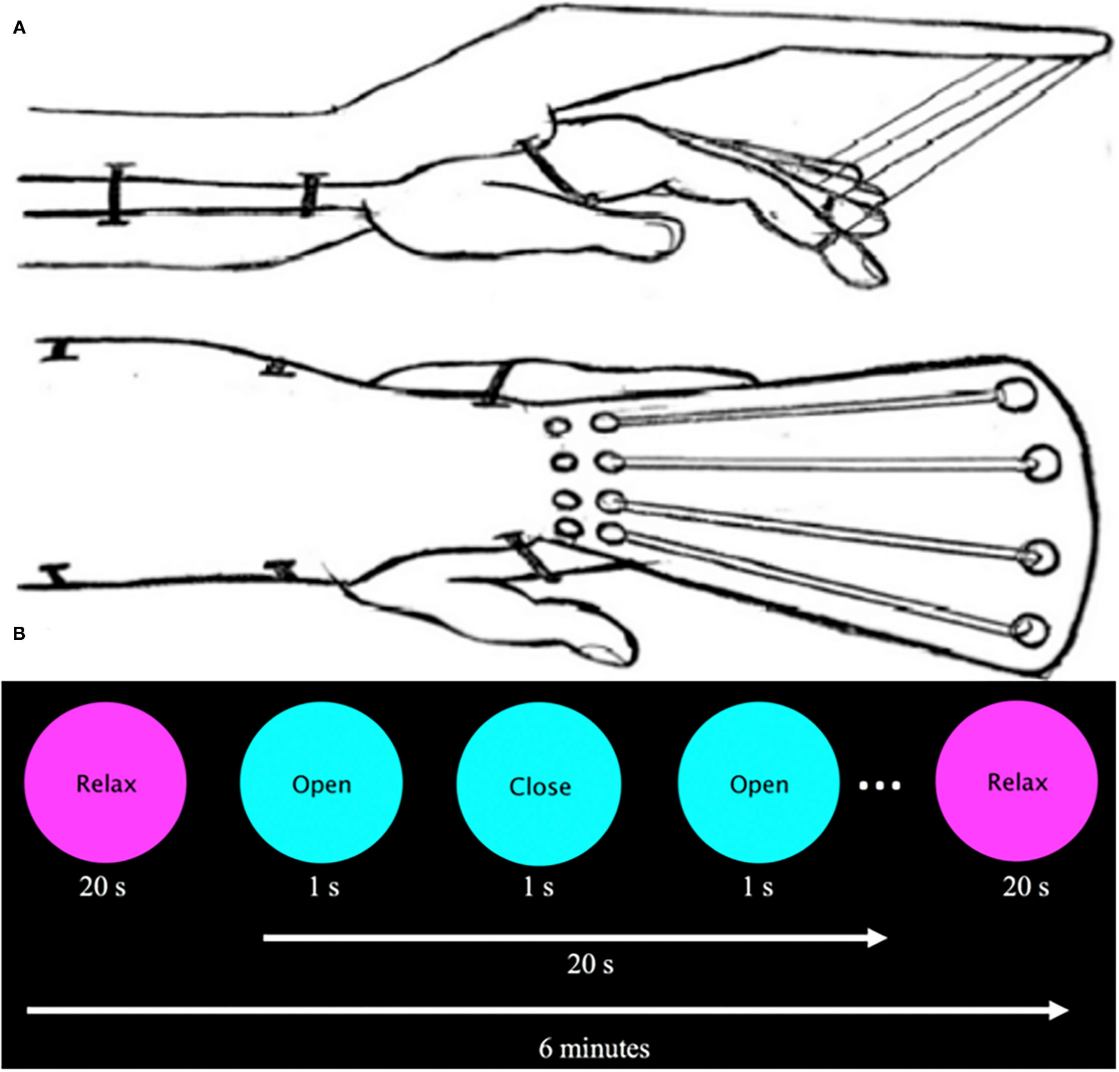
**(A)** Sketch of assistive finger extension device. This device assists finger extension of stroke participants with spasticity via elastic bands (Theraband: 10.7 N resistance at 100% elongation, 15.1 N at 200%) that are supported by an outrigger on the dorsal part of the hand. Velcro straps attached to the patient's first four digits and were connected to an elastic band. A plastic brace served to stabilize the participant's forearm and wrist as they were instructed to flex and extend the digits. **(B)** Visual presentation prompt. The visual prompt was presented on a screen inside the bore and read “Relax” centered in a magenta circle (20 s) and “Open/Close” centered on a cyan circle (20 s, 1 Hz) for 6 min.

Due to spasticity, some participants with stroke were unable to fully extend the fingers without assistance. To address this issue, a device was created to aid in finger extension and was used by all participants (stroke and control) during the task-based fMRI assessment. The device is described and depicted in Figure 2A. All participants actively flexed against the resistive bands while the device passively extended the fingers. In effect, while this device allowed for all subjects to complete the task, it also resulted in predominantly active finger flexion while producing passive finger extension. In addition, the device allowed testing within the spastic joint range of motion for the fingers (36). The substantial loss of cortical modulation of stretch reflex threshold (37) is likely to impact the extended finger range of motion, especially for the fingers. Since motor learning can be impaired in the spastic joint range of motion (38), the range of motion used for testing in this study was designed to include a pre-injection spastic range of motion that was relieved by BoNT-A injections.

Participants were scanned while prompted by a visual cue to perform full-hand flexion and extension using the affected (stroke group) or non-dominant (control group) hand. A visual cue was presented in a block paradigm, which alternated rest and hand movement at 20-s intervals for a total of 6 min (Figure 2B). Each participant performed two experimental runs.

### Data Pre-processing

The first four TRs were removed from each fMRI trial, and both trials were concatenated (39). Advanced Normalization Tools (ANTs) software (*N4BiasFieldCorrection*) corrected for bias field inhomogeneities in both anatomical and functional MR images (40). The skull and other non-brain matter were removed from both anatomical and functional MR images using the FMRIB Software Library (FSL) Brain Extraction Tool (*bet*) (41). Anatomical and functional MR images were flipped in the right-left direction for stroke participants with left hemiparesis and control participants self-identified as right-hand dominant, to standardize brain activation to the left hemisphere. In total, 5 stroke participants and 4 control participants were flipped. Registration to the Montreal Neurological Institute (MNI) 152-subject average brain was performed using ANTs for all images, and a non-linear warp was applied to the functional MR images. Lesion masks obtained from the Lesion Identification with Neighborhood Data Analysis (LINDA) algorithm (42) aided in image registration for stroke participants. Lesion masks and registration were visually inspected for accuracy.

### Activity Analysis

FMRI analyses were carried out using the fMRI Expert Analysis Tool (FEAT) Version 5.0, in FSL. First-level FEAT analysis was performed on individual data using a Fixed Effect (FE) analysis (43), which included motion correction, spatial smoothing using a full-width/half-maximum (FWHM) 5 mm Gaussian kernel, temporal high pass filter (0.01 Hz) on the BOLD signal, and prewhitening. A modeled hemodynamic response (HDR) was created by convolving the binary block design with a gamma wave (phase = 0s, std. dev. = 3s, mean lag = 6s), each voxel's timeseries was correlated to the time series model, and the resulting activation images were clustered and thresholded at a *Z*-value > 2.3 (*p* < 0.05).

Group analysis was performed using a general linear model (GLM) which categorized individual participants by their group (Stroke/Control) and session (W0/W6). Group mean activation maps were created using non-parametric permutation testing (10,000 permutations) for each group at a threshold *Z* > 2.3 and cluster significance *p* < 0.05 with false discovery rate correction.

### Regions of Interest for Activation

Within the stroke group, the activation images at time-point W6 were compared to the activation images at time-point W0, and the voxels where W6 was >W0 defined our volume of interest. These regions were further subdivided using the Jülich Histological Atlas for supratentorial regions and the Taliarch Daemon Label Atlas for cerebellar regions, and the number of active voxels and the intensity of each region was recorded.

### Regions of Interest for Connectivity

**Figure 3:**
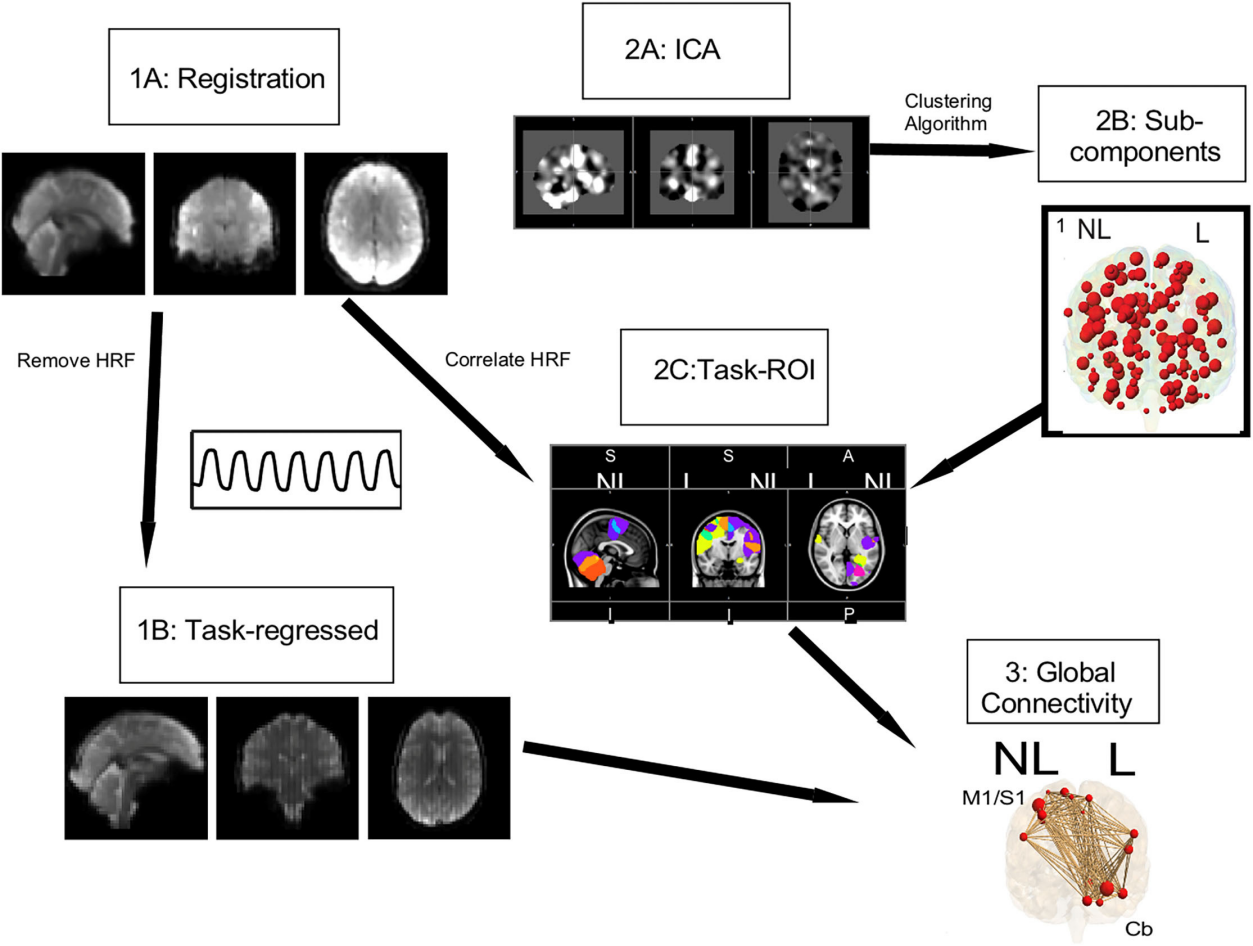
Connectivity pipeline. Registration (1A) and task regression (1B) were performed on the data collected for this study. Using the Smith et al. (44) data, an ICA (2A) and a clustering algorithm were performed to get 149 subcomponents, shown in red circles (2B). From these 149 subcomponents, task ROI (2C) were defined by correlating the HRF with the mean timeseries of our study's data. For these task ROI, shown with red circles the global connectivity (3) strength, shown with brown lines, was calculated.

For the connectivity analysis, ROIs were defined using publicly available data. First the ICA of these control data in both resting state and task-based functional connectivity was determined (44) (Figure 3,2A). These processes resulted in 149 sub-components (Figure 3,2B), in which only the gray matter volume was included within region of interest masks for the connectivity analysis. Utilizing these ROIs defined by functional connectivity instead of a priori ROIs allows for detection of the full functional connectivity network for a given region.

Using this study's task-based fMRI data, task ROIs were identified by correlating the hemodynamic response function (HRF) with the mean timeseries of each ROI as defined above (Figure 3,2C) [see Vinehout et al. (25) for additional details]. Briefly, subcomponents that were correlated with the HRF (*r* > 0.2) were considered task regions of interest (task ROIs). This was done across all stroke and control participants so one set of task-ROIs was defined for all participants in this study. See Table 3 for descriptions of the resulting ROIs. These task-ROIs corresponded to the ROIs involved in the finger flexion/extension task. This seed-based functional connectivity approach allows targeted assessment of motor pathways (45–47).

**Table 3:** Eighteen functional connectivity ROIs.

**ROI #**	**Main region of interest**	**Size**	**ROI**
		**(Voxels)**	**(X,Y,Z)**
1	Right Limbic Lobe and Cingulate Gyrus (BA 24)	1052	47, 57, 50
	Right Limbic Lobe and Cingulate Gyrus (BA 6)		
2	Right Anterior Lobe of Cerebellum (AlCb)	3332	55, 35, 20
3	Left Parietal Lobe and Inferior Parietal Lobule (BA 40)	7588	25, 50, 63
	Left Parietal Lobe and Postcentral Gyrus (BA 3)		
4	Right Frontal Lobe and Medial Frontal Gyrus (BA 6)	3039	50, 64, 66
5	Left Frontal Lobe and Superior Frontal Gyrus (BA 6)	1598	41, 64, 67
6	Right Frontal Lobe and Precentral Gyrus (BA 4)	4011	72, 60 50
	Right Frontal Lobe and Precentral Gyrus (BA 6)		
7	Left Frontal Lobe and Precentral Gyrus (BA 4)	3766	18, 59 49
	Left Frontal Lobe and Precentral Gyrus (BA 6)		
8	Right Anterior Lobe/Posterior of Cerebellum	1879	54 30 22
9	Left Parietal Lobe and Postcentral Gyrus (BA 3)	4279	38, 52, 69
	Left Frontal Lobe and Medial Frontal Gyrus (BA 6)		
10	Right Posterior/Anterior Lobe of Cerebellum. (PAlCb)	3879	59, 28, 28
11	Right Anterior/Posterior Lobe of Cerebellum (APlCb)	5197	49, 34, 21
12	Right Sub-lobar and Insula (BA 13)	4633	69, 53, 43
13	Right Anterior/Posterior Lobe of Cerebellum (PlCb)	5253	66, 31, 24
14	Left Parietal Lobe and Inferior Parietal Lobule (BA 40)	4187	27, 44, 57
15	Left Frontal Lobe and Middle Frontal Gyrus (BA 6)	4667	27, 60, 59
	Left Frontal Lobe and Precentral Gyrus (BA 6)		
16	Left Parietal Lobe and Postcentral Gyrus (BA 5)	1401	30, 43, 69
	Left Parietal Lobe and Inferior Parietal Lobule (BA 40)		
17	Right Anterior Lobe of Cerebellum (AlCb)	8012	58, 42, 26
18	Right Anterior/Posterior Lobe of Cerebellum (APlCb)	4894	49, 27, 28

*Table listing all the task ROI derived from the group ICA of controls. The number of voxels correspond to the size of each ROI in 2mm standard MNI space. X, Y, Z coordinates refer to standard MNI space. Percentages listed are for each gray matter region that encompassed more than 5% of a given ROI. Names provided are based on the labels of the Talairach Atlas. BA, Brodmann's Area*.

### Functional Connectivity Analysis

After identification of task-ROIs, the HRF of the flexion/extension task was regressed out of the task-based fMRI data. The HRF was removed for task-based functional connectivity to reduce the effect that brain activations have on spurious connectivity measurements (48, 49). For each participant and task, a mean fMRI time series was computed for each of the 18 identified task ROIs (see Table 3 for location of task-ROIs). Pearson correlation coefficients were computed on all pairwise combinations of this mean time series for the 18 task ROIs; Fisher-Z transformations were applied to the Pearson correlation coefficients. These values provided a measure of global connectivity that represented the strength of functional connections between task ROIs (Figure 3,3). The FSL randomize (41) non-parametric permutation test with Bonferroni correction for multiple comparisons was used for comparisons between W0 and W6. These measures provided insight into the strength of the functional connections among task ROIs.

### Correlation With Clinical Impairment

Correlations were performed between activation volume, activation intensity, functional connectivity, and clinical measurements, using a Spearman correlation for MAS and a Pearson correlation for FMA. These correlations were performed for W0 and W6 measurements, corrected for multiple comparisons with a False Discovery Rate.

## Results

### Reduced Motor Impairment Following BoNT-A Therapy

Stroke participants showed a significant increase in FMA motor scores following injections of BoNT-A to the affected arm (*p* = 0.004; *df* = 8; *t*-stat = −3.9194; paired *t*-test) with a mean increase of 2.1 ± 1.62 (Table 1). Four of nine participants showed improvement in wrist function, and three showed improvements in finger extension. Those that improved in finger extension had the highest MAS scores prior to injection. Other areas of improvement (mass finger extension, forearm pronation/supination, shoulder flexion, and abduction) varied between participants.

### Changes in Brain Activity Patterns Following BoNT-A Therapy

**Figure 4:**
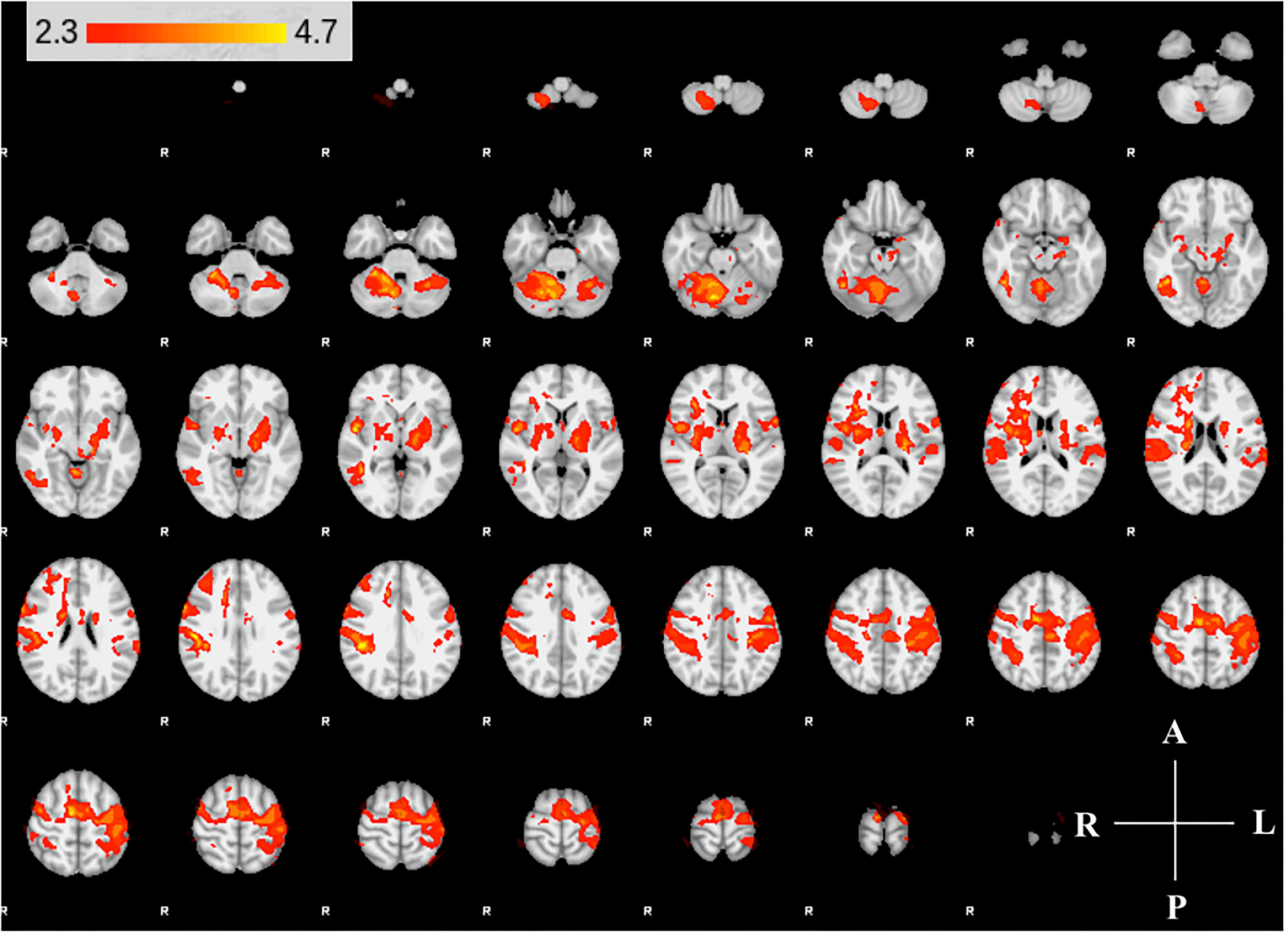
Group activity maps of control participants. The figure shows slices of the MNI template overlaid with z-statistic (*Z* > 2.3) maps of W0 and W6 average control group activity during non-dominant hand movement. Activity maps indicate volumes in which there was significant (*p* < 0.05) levels of activity across the group. The right hemisphere of the brain is displayed on the left. Right hemisphere is ipsilateral to the movement arm. No differences were detected between W0 and W6.

**Figure 5:**
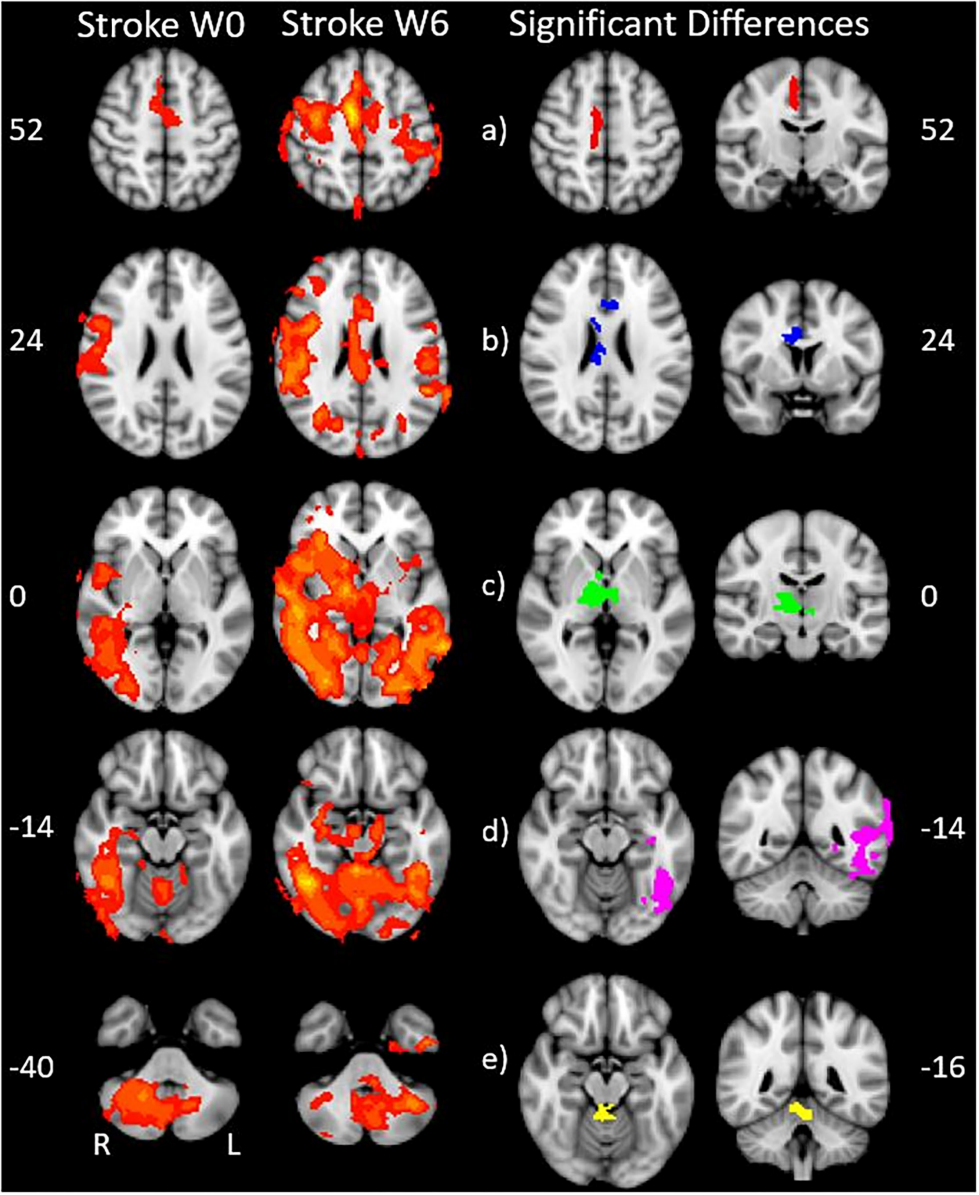
Stroke differences during BoNT-A injections. The first two columns show significant (*p* < 0.05) levels of activity across the stroke group for W0 (left) and W6 (right). Columns 3 and 4 show the coronal (left) and axial (right) view of significant increase in activity following the BoNT-A intervention, displayed as five regions of interests. These areas are: **(a)** right premotor cortex **(b)** right cingulate gyrus **(c)** right thalamus **(d)** sensory integration area, **(e)** superior cerebellum. The physical coordinates of the axial slices are shown to the left or right of axial images, and the coronal views are denoted by the yellow horizontal lines.

We found expected activity patterns and no significant difference between W0 and W6 sessions for controls. Across sessions, controls consistently and significantly activated bilateral primary motor (M1) and ipsilateral cerebellar areas at W0 and W6 (Figure 4) during right-hand movement. In addition to these regions, the ipsilateral premotor and supplementary motor area, bilateral hand portion of the M1, the thalamus and the putamen showed significant task-related activity. Although control participant's activity maps were similar across sessions, stroke participants showed differences between W0 and W6 (Figure 5). At W6 there was more widespread and bilateral activation in the stroke group compared with W0; whereas activation before injection was restricted to the contralesional hemisphere, activation increased in both hemispheres after injection (Figure 5). Activation maps yielded *p*-values for each voxel; these maps were threshold with *p* < 0.05 to assess significance. Significant differences (*p* < 0.05, *df* = 8; *z* > 2.3; paired *z*-test) between W6 and W0 in the stroke group included: (1) contralesional premotor cortex (PMC-R), (2) contralesional cingulate gyrus (CG-R), (3) contralesional thalamus (Th-R), (4) somatosensory and visual integration areas (Sens-IA), and (5) superior cerebellum (S-CB). These regions of activation are further described in Table 4 and illustrated in Figure 5.

**Table 4:** Activation ROI characteristics.

**Main Region of Interest**	**Abbreviation**	**Size (Voxels)**	**Region description**	**ROI COG**
	PMC-R	441.00	64% GM Premotor Cortex BA6 R	X = 49.5, Y = 56.3, Z = 60.2
Premotor cortex			14% GM Primary Motor Cortex BA4a R	
	CG	758.00	40% WM Cingulum R	X = 46.9, Y = 58.3, Z = 49
Cingulate gyrus			18% WM Callosal Body	
	Th-R	855.00	78% Right Thalamus	X = 50.9, Y = 58.5, Z = 37.7
Right thalamus			19% Right Cerebral, WM	
	S-CB	298.00	Right Cerebellum	
Superior cerebellum			Anterior Lobe	X = 45.1, Y = 45.5, Z = 29.6
			Cerebellar Lingual	
	Sens-IA	2680.00	55% Inferior Temporal Gyrus, temporo-occipital part;	X = 20.7, Y = 36.4, Z = 36.4
Sensory integration area			14% Temporal Occipital Fusiform Cortex	
			7% Lateral Occipital Cortex, inferior division	
			3% Occipital Fusiform Gyrus	

*Probabilities describing each ROI's anatomical makeup were determined using Jülich Histological Atlas for cortical ROIs and the Taliarch Daemon Label Atlas for at the voxel of greatest z-score overlaid onto a 2 mm-MNI brain*.

*WM, White matter; GM, Gray matter*.

**Figure 6:**
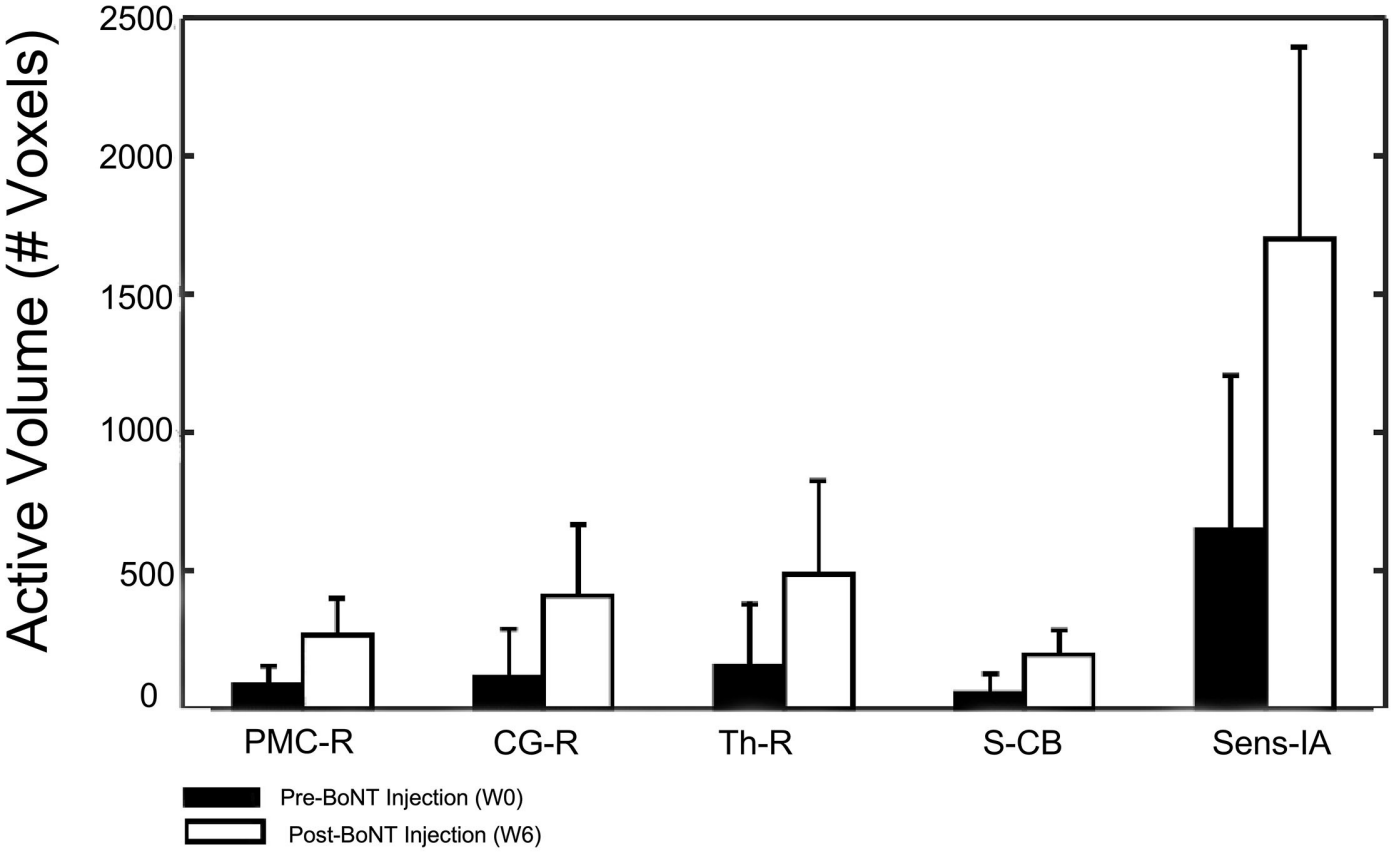
Changes in activation volume following BoNT-A injection therapy. Control group results showed no difference between sessions and are not included in this figure.

The five identified regions of activation were subsequently used as masks to identify the number of active voxels in the given volume for the stroke group at W0 and W6 (Figure 6). Participants with stroke showed a significantly increased number of active voxels in all five regions following BoNT-A injections.

### Changes in Brain Connectivity Following BoNT-A Therapy

**Figure 7:**
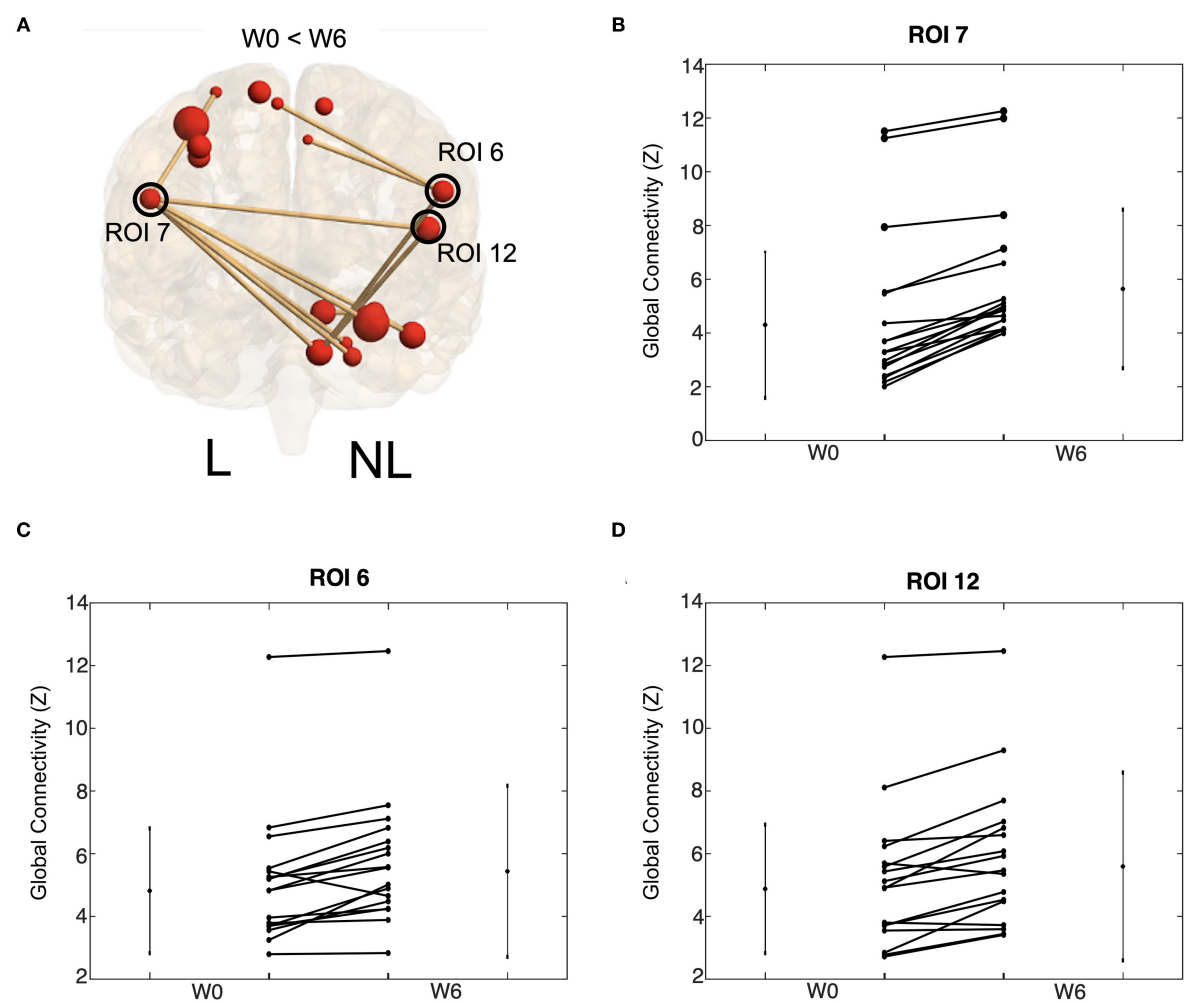
Increases tend in functional connectivity following BoNT-A injection therapy. Global connectivity strength for all task ROIs connected to ROI 7, 6, and 12. Visual depiction of these ROI **(A)**. The size of the red ball represents the size of the ROI and the thickness of the line references the Z-transformed correlation coefficient size. Only significant connections are visualized. Trends of ROI 7 **(B)**, ROI 6 **(C)**, and ROI 12 **(D)** are shown. These trends are for all task ROI connections to the given ROI; each line represents group average for one connection. Values are shown for W0 and W6 with a line connecting values of the same connection strength. Lines on the side of the graph show the mean and standard deviation of the connection strength across stroke participants.

Interestingly, the connectivity analysis did not show significant differences between W0 and W6 in either stroke or control groups after multiple-comparison corrections. Trends of increased functional connectivity after BoNT-A were observed across the 18 task-related ROIs; however, these trends were not significant once corrected for multiple comparisons. The largest connectivity changes were observed in three nodes, the bilateral premotor/motor cortices and the insula of the non-lesioned hemisphere (Figure 7 and Table 3), which showed small *p*-values but were not statistically significant when the correction for multiple comparisons was applied. In contrast, the control group did not demonstrate trends for increased functional connectivity between W0 and W6. See Figure 7 for visual depiction of trends in stroke participants for these 3 nodes.

### Clinical Correlations With Activity and Connectivity Values

**Figure 8:**
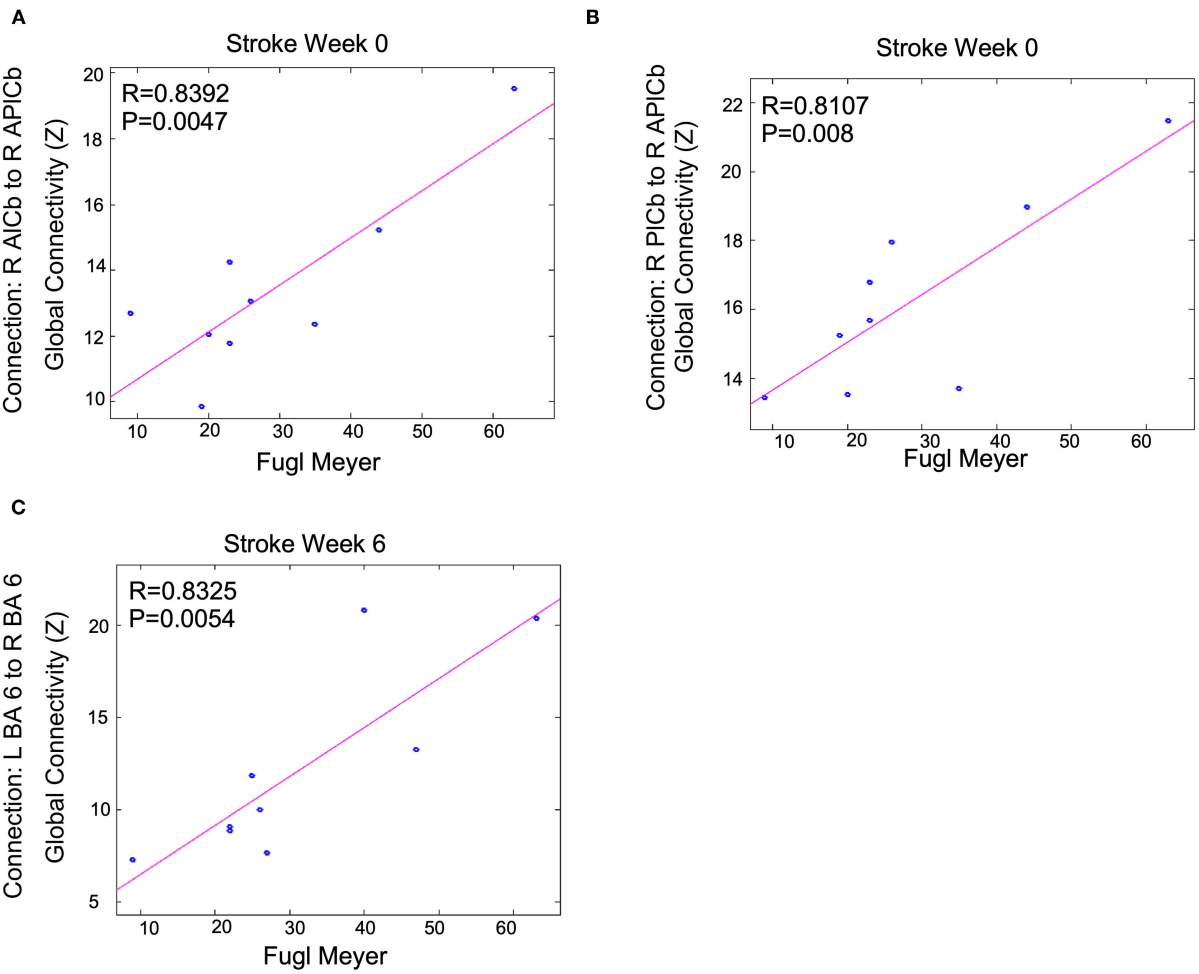
Functional connectivity measurements correlated with FMA scores following BoNT-A injection therapy: trends are shown above. Shown *P*-values are without correction for multiple comparisons across all tests run for all connections and clinical measurements. We only show the largest trends with these uncorrected *p* < 0.01. R is the correlation coefficient. R, right; L, left; BA, Brodmann's Area; A, Anterior; P, Posterior; lCb, lobule of the cerebellum. This shows Fugl Meyer scores correlation to **(A)** the connection between R AlCb to R APlCb, **(B)** the connection between R PlCb and R APlCb, and **(C)** the connection between L BA 6 and R BA 6.

Correlations were performed between activation volume, activation intensity, functional connectivity, and clinical measurements (MAS and FMA). The correlations between the MAS (W0) and activation volume, activation intensity and functional connectivity were not significant when corrected for multiple comparisons. There were trends between these measurements that had small *p*-values before multiple comparison correction. A total of 52 correlations had an uncorrected *p* < 0.05. Three correlations had uncorrected *p* < 0.01. These trends were W0 functional connectivity measurements that correlated with W0 FMA scores and W6 functional connectivity measurements that correlated with W6 FMA scores, as summarized in Figure 8. At W0, connections between the contralesional anterior cerebellum and the contralesional posterior cerebellum were moderate and positively correlated (*R* = 0.83 and *R* = 0.81) with W0 FMA scores. At W6, contralesional and ipsilesional premotor areas were moderate and positively (*R* = 0.83) correlated with the FMA scores.

## Discussion

In this study, we found preliminary evidence of the effects of BoNT-A on higher-order brain activation using fMRI. Following BoNT-A, significant increases in the BOLD signal in the stroke group were observed in the contralesional premotor cortex (PMC-R), cingulate gyrus (CG-R), and motor thalamus (Th-R), ipsilesional sensory integration regions (Sens-IA), and bilateral superior cerebellum (S-CB). These regions showed increased activity, characterized by both larger volume of activation and greater correlation to the HDR. Some connections between these areas were also correlated with the FMA scores. These results suggest that in people with spasticity, BoNT-A enables activation of higher motor centers, possibly associated with renewed access to networks associated with motor planning and control of movement.

### Increased Volume of Activation in Higher-Order Brain Regions After BoNT-A Therapy

Our functional activation results showed that BoNT-A therapy increased functional activity in the ipsilesional and contralesional hemispheres during unilateral paretic finger flexion/extension, suggesting BoNT-A therapy promotes neural reorganization. The activation patterns for the control group (Figure 4) were consistent with previous studies, showing activation in the contralateral motor areas in addition to the ipsilateral cerebellum (50–52). Interestingly, the control group also had activation of ipsilateral (right hemisphere) motor areas and bilateral subcortical regions, which have been associated with task precision and movement duration (53–56). In the stroke group, BoNT-A therapy increased the volume of activation in both hemispheres. Prior to injection (W0), stroke participants had activity associated with wrist flexion mainly in the contralesional hemisphere (Figure 5). After injection (W6), the volume of activation encompassed bilateral motor areas, subcortical regions including the thalamus, and the cerebellum, similar to the control group.

There are a limited number of studies on the effects of BoNT-A therapy on brain activation. Contrary to our results, reduced brain activation is observed after BoNT-A therapy for imagined movements (57), finger tapping (19), and mass finger flexion (17). In particular, Manganotti et al. (17) observed decreased BOLD activation following BoNT-A injection therapy, localizing in the ipsilesional motor cortex and contralesional cerebellum. There are two key methodological differences in our study and these prior studies. First, our protocol used a device that passively extended the fingers, which facilitated finger movement through the full range; prior studies involved paced isotonic contractions, which were constrained by an orthosis allowing for 30° range of motion. The second key difference is our protocol aimed to observe the effects of BoNT-A injections on brain activity during the normal course of stroke rehabilitation.

The increase in brain activation following BoNT-A injection therapy in stroke participants might be due to an increased neural drive to flex the fingers through the full range of motion, or, at least in some participants, because physical therapy contributes to the increase in activation. In a similar BoNT-A and fMRI study, Diserens et al. (21) found bilateral increases in BOLD activation in motor regions following BoNT-A injections during passive arm movement, and the BOLD activity increased further following paced repetitive passive movements of the plegic hand and BoNT-A injections. Additionally, Veverka et al. (20) found increased volume of activation in the bilateral cerebellum, contralesional sensorimotor cortex, and the contralesional occipital cortex during passive wrist movements. Thus, reducing spastic hypertonia may increase the afferent feedback from hand movement (20, 21) and allow for greater improvements during physiotherapy (58–60), resulting in increased brain activation.

Another possible reason we saw differences in activation in bilateral brain regions compared to prior studies might be differences in the stroke participant pre-injection function. The participants in our study had heterogeneity of their baseline Fugl-Meyer scores ranging from 9 to 63. The inclusion of high impairment participants is reflected in these participants with lower Fugl-Meyer scores. Stroke survivors have increased activation, particularly in the contralesional hemisphere, during hand movements (13, 61–64). Additionally, it has been reported that therapy increases activity in the ipsilesional hemisphere, lateralizing and localizing activity during paretic hand movement (65–68). Our results included an increased ipsilesional activation following BoNT-A injection therapy, which has been associated with improved motor recovery (69, 70). However, our results also showed increased activation in the contralesional side. It has been suggested that hyper-excitability of the contralesional hemisphere is detrimental to motor recovery following stroke due to interhemispheric inhibition (71). However, recent findings suggest that well-recovered patients have increased contralesional motor activity, which may play a supportive role during rehabilitation (71, 72). This trend might be more apparent in study participants with severe motor deficits (13, 14, 66).

### Functional Connectivity in Motor-Related Regions After BoNT-A Therapy

While there were no significant differences in connectivity between W0 and W6 for the stroke or control participants, there was a trend of increased connectivity in the stroke group at W6. A larger sample size might have been able to parse out these differences. Changes in functional connectivity after treatment can be significant (73–75). During W0 we saw correlations between regions of the right cerebellum and FMA score; during W6 we saw correlations in the connectivity between the contralesional and ipsilesional premotor areas and FMA score. Interestingly the areas that overlapped between functional connectivity and activation were areas in which functional connectivity correlated with the FMA score. This further highlights the importance of the cerebellum and contralesional and ipsilesional premotor areas in recovery. The W6 correlations between contralesional and ipsilesional premotor areas suggest that these connections might be more clinically relevant as people with stroke recover. Others have highlighted the clinical importance of these interhemispheric connections (76).

### Study Limitations

The study enrollment was small, and it is possible that including more participants would have provided more areas of significant activity and connectivity differences with BoNT-A therapy. However, despite the low sample size, there were consistent patterns within the stroke group. A larger sample size might have provided significant functional connectivity results after correction for multiple comparisons, verifying the observed trends. This sample included a heterogeneous group of stroke patients. Arm therapy decreases brain activation in stroke survivors with high baseline function and increases brain activity in those with low initial function (77), suggesting that the variability of stroke severity in our test group might have limited the statistical significance of group comparisons. In the future, a larger sample size could be collected to assess how stroke severity affects changes in brain activation following BoNT-A therapy.

Another limitation to the present study is the lack of measurement of hand movement while in the scanner. Movement of the target had was observed visually in all participants in the current study during fMRI measurements; however, a real-time measurement of hand movement would provide a measure of the change in movement between W0 and W6, which could have impacted brain activity. The hand apparatus helped to normalize movement across participants and sessions, although differences in movement range were still possible. In addition, measurements of movements would allow for control of mirror movements that are often seen in stroke survivors trying to perform tasks with a significantly impaired limb (78–80). It is possible that activity seen in contralesional motor areas may have resulted from mirror movements of the unaffected hand, though it is unlikely because mirror movements were not observed in the orientation sessions.

Assessments of improvements in spasticity were limited to the MAS and FMA prior to the injection. In addition, MAS is a measurement of spastic muscle tone and does not distinguish active and passive components of hypertonia. A follow-up measure to quantify improvements in spastic hypertonia and impairment would have added to the interpretation of the BoNT-A effects. Additional tests of hand function would have added information on functional ability following BoNT-A therapy. We identified a significant increase of 2.1 FMA points across the group, which is a small increase in the upper extremity FMA, compared to the reported 3.2 minimal detectable change in individual upper extremity score (34). While we saw significant changes in FMA scores pre and post BoNT-A injection (Table 1), these changes were below minimal clinically important differences for this scale (81). Assessments that directly measure the functional goals targeted by the BoNT-A therapy might be more meaningful for future work.

Physical therapy was recommended to patients participating in this study as part of their standard clinical care, with exercises tailored to their specific needs. Physical therapy treatment plans, duration, and frequency were not controlled, but assumed to remain constant over the 6-week period of involvement in the study. For a number of practical reasons, participation in physical therapy sessions was limited (see Table 1 for therapy recommendations and Table 2 for the number of therapy sessions completed by each participant). Thus, the contribution of therapy to the changes in activity and connectivity following BoNT-A injections remains unclear. Injection site and BoNT-A dose were determined by the severity of spasticity and clinical need of each participant. The number of BoNT-A injections that each participant received prior to the study period ranged from 2 to 40. None of these variables correlated with the study outcomes. However, because this study did not include participants after their initial BoNT-A injections the results may not have captured the most significant improvements. Greater improvements in function have been reported following the initial BoNT-A injection (82–84) and subsequent injections are needed to maintain those improvements.

## Conclusion

This study showed the effects BoNT-A injection therapy on motor impairment and neuroplasticity. BoNT-A injection therapy produced a significant increase in contralesional activation in stroke survivors after therapy. Additionally, there was a trend of increased interhemispheric and intrahemispheric functional connectivity, most notably to motor/premotor nodes. These neuroplastic changes correlated with motor impairment and limb spasticity; ipsilesional functional connectivity measurements were correlated with the Fugl-Meyer scores, and ipsilesional activation measurements were correlated with the Modified Ashworth Scale. These results suggest that neuroplastic effects take place in response to improvements in focal spasticity and highlight the importance of brain activity and connectivity patterns in rehabilitation.

## Data Availability Statement

The raw data supporting the conclusions of this article will be made available by the authors, without undue reservation.

## Ethics Statement

The studies involving human participants were reviewed and approved by Medical College of Wisconsin: PRO00027569. The patients/participants provided their written informed consent to participate in this study.

## Disclosure

The presented work has not been published prior, although this work constitutes a portion of the Master of Science thesis of KT.

## Author Contributions

BS, KT, and AH contributed to the study conception and design. MS and KT contributed to the material preparation and data collection. Analyses were performed by KT and KV. The first draft of the manuscript was written by KT and KV. All authors commented on previous versions of the manuscript, read and approved the final manuscript.

## Funding

Funding was provided by the Strategic Fund, a component of the Advancing a Healthier Wisconsin endowment at the Medical College of Wisconsin.

## Conflict of Interest

The authors declare that the research was conducted in the absence of any commercial or financial relationships that could be construed as a potential conflict of interest.

## Publisher's Note

All claims expressed in this article are solely those of the authors and do not necessarily represent those of their affiliated organizations, or those of the publisher, the editors and the reviewers. Any product that may be evaluated in this article, or claim that may be made by its manufacturer, is not guaranteed or endorsed by the publisher.

## References

[1] Watkins CL Leathley MJ Gregson JM Moore AP Smith TL Sharma AK. Prevalence of spasticity post stroke. Clin Rehabil. (2002) 16:515–22. 10.1191/0269215502cr512oa 12194622

[2] Wissel J Manack A Brainin M. Toward an epidemiology of poststroke spasticity. Neurology. (2013) 80:S13–S19. 10.1212/WNL.0b013e3182762448 23319481

[3] Esquenazi A Wein TH Ward AB Geis C Liu C Dimitrova R. Optimal muscle selection for onabotulinumtoxina injections in poststroke lower-limb spasticity: a randomized trial. Am J Phys Med Rehabil. (2019) 98:360–8. 10.1097/PHM.0000000000001101 31003229

[4] Slawek J Bogucki A Reclawowicz D. Botulinum toxin type A for upper limb spasticity following stroke: an open-label study with individualised, flexible injection regimens. Neurol Sci. (2005) 26:32–9. 10.1007/s10072-005-0379-8 15877185

[5] Esquenazi A Albanese A Chancellor MB Elovic E Segal KR Simpson DM. Evidence-based review and assessment of botulinum neurotoxin for the treatment of adult spasticity in the upper motor neuron syndrome. Toxicon. (2013) 67:115–28. 10.1016/j.toxicon.2012.11.025 23220492

[6] Pandyan AD Price CI Barnes MP Johnson GR. A biomechanical investigation into the validity of the modified Ashworth Scale as a measure of elbow spasticity. Clin Rehabil. (2003) 17:290–4. 10.1191/0269215503cr610oa 12735536

[7] Teasell R Foley N Pereira S Sequeira K Miller T. Evidence to practice: botulinum toxin in the treatment of spasticity post stroke. Top Stroke Rehabil. (2012) 19:115–21. 10.1310/tsr1902-115 22436359

[8] Bhakta BB Cozens JA Bamford JM Chamberlain MA. Use of botulinum toxin in stroke patients with severe upper limb spasticity. J Neurol Neurosurg Psychiatry. (1996) 61:30–5. 10.1136/jnnp.61.1.30 8676154 PMC486452

[9] Shaw L Rodgers H Price C van Wijck F Shackley P Steen N. BoTULS: a multicentre randomised controlled trial to evaluate the clinical effectiveness and cost-effectiveness of treating upper limb spasticity due to stroke with botulinum toxin type A. Health Technol Assess. (2010) 14:1–113. 10.3310/hta14260 20515600

[10] Childers MK Brashear A Jozefczyk P Reding M Alexander D Good D. Dose-dependent response to intramuscular botulinum toxin type A for upper-limb spasticity in patients after a stroke. Arch Phys Med Rehabil. (2004) 85:1063–9. 10.1016/j.apmr.2003.10.015 15241751

[11] Ward AB Wissel J Borg J Ertzgaard P Herrmann C Kulkarni J and Best Study Group. Functional goal achievement in post-stroke spasticity patients: the BOTOX® Economic Spasticity Trial (BEST). J Rehabil Med. (2014) 46:504–13. 10.2340/16501977-1817 24715249

[12] Wissel J Bensmail D Ferreira JJ Molteni F Satkunam L Moraleda S. Safety and efficacy of incobotulinumtoxinA doses up to 800 U in limb spasticity: the TOWER study. Neurology. (2017) 88:1321–8. 10.1212/WNL.0000000000003789 28283596 PMC5379931

[13] Cramer SC Nelles G Benson RR Kaplan JD Parker RA Kwong KK. A functional MRI study of subjects recovered from hemiparetic stroke. Stroke. (1997) 28:2518–27. 10.1161/01.STR.28.12.2518 9412643

[14] Ward NS Brown MM Thompson AJ Frackowiak R. Neural correlates of outcome after stroke: a crossl correlat fMRI study. Brain. (2003) 126:1430–48 10.1093/brain/awg145 12764063 PMC3717456

[15] Rehme AK Fink GR von Cramon DY Grefkes C. The role of the contralesional motor cortex for motor recovery in the early days after stroke assessed with longitudinal FMRI. Cereb Cortex. (2011) 21:756–68. 10.1093/cercor/bhq140 20801897

[16] Bergfeldt U Jonsson T Bergfeldt L Julin P. Cortical activation changes and improved motor function in stroke patients after focal spasticity therapy–an interventional study applying repeated fMRI. BMC Neurol. (2015) 15:52. 10.1186/s12883-015-0306-4 25884323 PMC4450484

[17] Manganotti P Acler M Fiaschi A Formaggio E Mucelli RP Cerini R. Changes in cerebral activity after decreased upper-limb hypertonus: an EMG-fMRI study. Magn Reson Imaging. (2010) 28:646–52. 10.1016/j.mri.2009.12.023 20117894

[18] Tomášová Z Hluštík P Král M Otruba P Herzig R Krobot A. Cortical activation changes in patients suffering from postical ac arm spasticity and treated with botulinum toxin A. J Neuroimaging. (2013) 23:337–44. 10.1111/j.1552-6569.2011.00682.x 22212022

[19] Veverka T Hluštík P Hok P Otruba P Tüdös Z Zapletalová J. Cortical activity modulation by botulinum toxin type A in patients with post-stroke arm spasticity: real and imagined hand movement. J Neurol Sci. (2014) 346:276–83. 10.1016/j.jns.2014.09.009 25255982

[20] Veverka T Hluštík P Hok P Otruba P Zapletalová J Tüdös Z. Sensorimotor modulation by botulinum toxin A in post-stroke arm spasticity: passive hand movement. J Neurol Sci. (2016) 362:14–20 10.1016/j.jns.2015.12.049 26944111

[21] Diserens K Ruegg D Kleiser R Hyde S Perret N Vuadens P. Effect of repetitive arm cycling following botulinum toxin injection for poststroke spasticity: evidence from fMRI. Neurorehabil Neural Repair. (2010) 24:753–62 10.1177/1545968310372138 20663964

[22] Carter AR Patel KR Astafiev SV Snyder AZ Rengachary J Strube MJ. Upstream dysfunction of somatomotor functional connectivity after corticospinal damage in stroke. Neurorehabil Neural Repair. (2012) 26:7–19. 10.1177/1545968311411054 21803932 PMC3822763

[23] Kalinosky BT Berrios Barillas R Schmit BD. Structurofunctional resting-state networks correlate with motor function in chronic stroke. Neuroimage Clin. (2017) 16:610–23. 10.1016/j.nicl.2017.07.002 28971011 PMC5619927

[24] Kalinosky BT Vinehout K Sotelo MR Hyngstrom AS Schmit BD. Tasked-based functional brain connectivity in multisensory control of wrist movement after stroke. Front Neurol. (2019) 10:609. 10.3389/fneur.2019.00609 31263444 PMC6585311

[25] Vinehout K Schmit BD Schindler-Ivens S. Lower limb task-based functional connectivity is altered in stroke. Brain Connect. (2019) 9:365–77. 10.1089/brain.2018.0640 30799641 PMC6909701

[26] Westlake KP Nagarajan SS. Functional connectivity in relation to motor performance and recovery after stroke. Front Syst Neurosci. (2011) 5:8. 10.3389/fnsys.2011.00008 21441991 PMC3060711

[27] Fugl-Meyer AR Jääskö L Leyman I Olsson S Steglind S. The post-stroke hemiplegic patient. 1. a method for evaluation of physical performance. Scand J Rehabil Med. (1975) 7:13–31. 1135616

[28] Bohannon RW Smith MB. Interrater reliability of a modified ashworth scale of muscle spasticity. Phys Ther. (1987) 67:206–7. 10.1093/ptj/67.2.206 3809245

[29] Gregson JM Leathley MJ Moore AP Smith TL Sharma AK Watkins CL. Reliability of measurements of muscle tone and muscle power in stroke patients. Age Ageing. (2000) 29:223–8. 10.1093/ageing/29.3.223 10855904

[30] Kaya T Karatepe AG Gunaydin R Koc A Ercan UA. Inter-rater reliability of the Modified Ashworth Scale and modified Modified Ashworth Scale in assessing poststroke elbow flexor spasticity. Int J Rehabil Res. (2011) 34:59–64. 10.1097/MRR.0b013e32833d6cdf 20671560

[31] Katz RT Rove GP Brait C Rymer WZ. Objective quantification of spastic hypertonia: correlation with clinical findings. Arch Phys Med Rehabil. (1992) 73:339–47. 10.1016/0003-9993(92)90007-J 1554307

[32] Duncan PW Propst M Nelson SG. Reliability of the Fugl-Meyer assessment of sensorimotor recovery following cerebrovascular accident. Phys Ther. (1983) 63:1606–10. 10.1093/ptj/63.10.1606 6622535

[33] Sullivan KJ Tilson JK Cen SY Rose DK Hershberg J Correa A. Fugl-Meyer assessment of sensorimotor function after stroke: standardized training procedure for clinical practice and clinical trials. Stroke. (2011) 42:427–32. 10.1161/STROKEAHA.110.592766 21164120

[34] See J Dodakian L Chou C Chan V McKenzie A Reinkensmeyer DJ. A standardized approach to the Fugl-Meyer assessment and its implications for clinical trials. Neurorehabil Neural Repair. (2013) 27:732–41. 10.1177/1545968313491000 23774125

[35] Murphy MA Resteghini C Feys P Lamers I. An overview of systematic reviews on upper extremity outcome measures after stroke. BMC Neurol. (2015) 15:29. 10.1186/s12883-015-0292-6 25880033 PMC4359448

[36] Kamper DG Schmit BD Rymer WZ. Effect of muscle biomeckamhanics on the quantification of spasticity. Ann Biomed Eng. (2001) 29:1122–34. 10.1114/1.1424918 11853265

[37] Piscitelli D Turpin NA Subramanian SK Feldman AG Levin MF. Deficits in corticospinal control of stretch reflex thresholds in stroke: implications for motor impairment. Clin Neurophysiol. (2020) 131:2067–78. 10.1016/j.clinph.2020.05.030 32682234

[38] Subramanian SK Feldman AG Levin MF. Spasticity may obscure motor learning ability after stroke. J Neurophysiol. (2018) 119:5–20. 10.1152/jn.00362.2017 28904099 PMC5866466

[39] Diedrichsen J Shadmehr R. Detecting and adjusting for artifacts in fMRI time series data. Neuroimage. (2005) 27:624–34. 10.1016/j.neuroimage.2005.04.039 15975828 PMC1479857

[40] Avants BB Tustison N Song G Cook PA Klein A Gee JC. A reproducible evaluation of ANTs similarity metric performance in brain image registration. Neuroimage. (2011) 54:2033–44. 10.1016/j.neuroimage.2010.09.025 20851191 PMC3065962

[41] Jenkinson M Beckmann CF Behrens TEJ Woolrich MW Smith SM. FSL. Neuroimage. (2012) 62:782–90. 10.1016/j.neuroimage.2011.09.015 21979382

[42] Pustina D Coslett HB Turkeltaub PE Tustison N Schwartz MF Avants B. Automated segmentation of chronic stroke lesions using LINDA: lesion identification with neighborhood data analysis. Hum Brain Mapp. (2016) 37:1405–21. 10.1002/hbm.23110 26756101 PMC4783237

[43] Woolrich MW Ripley BD Brady M Smith SM. Temporal autocorrelation in univariate linear modeling of FMRI data. Neuroimage. (2001) 14:1370–86 10.1006/nimg.2001.0931 11707093

[44] Smith SM Fox PT Miller KL Glahn DC Fox PM Mackay CE. Correspondence of the brain's functional architecture during activation and rest. Proc Natl Acad Sci. (2009) 106:13040–5. 10.1073/pnas.0905267106 19620724 PMC2722273

[45] Biswal B Zerrin Yetkin F Haughton VM Hyde JS. Functional connectivity in the motor cortex of resting human brain using echotional MRI. Magn Reson Med. (1995) 34:537–41. 10.1002/mrm.1910340409 8524021

[46] Lee JY Choi Y Ahn KJ Nam Y Jang JH Choi HS. Seed-based resting-state functional MRI for presurgical localization of the motor cortex: a task-based functional MRI-determined seed versus an anatomy-determined seed. Korean J Radiol. (2019) 20:171–9. 10.3348/kjr.2018.0004 30627033 PMC6315064

[47] Gandhi TK. Resting state fMRI analysis using seed based and ICA methods. In: 2016 3rd International Conference on Computing for Sustainable Global Development (INDIACom). (2016). p. 2551–4. IEEE.

[48] Cole MW Ito T Schultz D Mill R Chen R Cocuzza C. Task activations produce spurious but systematic inflation of task functional connectivity estimates. Neuroimage. (2019) 189:1–18. 10.1016/j.neuroimage.2018.12.054 30597260 PMC6422749

[49] Rangaprakash D Wu GR Marinazzo D Hu X Deshpande G. Hemodynamic Response Function (HRF) variability confounds restingariabi fMRI functional connectivity. Magn Reson Med. (2018) 80:1697–713. 10.1002/mrm.27146 29656446

[50] Meier JD Aflalo TN Kastner S Graziano MS. Complex organization of human primary motor cortex: a high-resolution fMRI study. J Neurophysiol. (2008) 100:1800–12 10.1152/jn.90531.2008 18684903 PMC2576195

[51] Olman CA Pickett KA Schallmo M Kimberley TJ. Selective BOLD responses to individual finger movement measured with fMRI at 3T. Hum Brain Mapp. (2012) 33:1594–606 10.1002/hbm.21310 21674691 PMC3713710

[52] Yoo S Wei X Dickey CC Guttmann CR Panych LP. Long-term reproducibility analysis of fMRI using hand motor task. Int J Neurosci. (2005) 115:55–77 10.1080/00207450490512650 15768852

[53] Bernard RA Goran DA Sakai ST Carr TH McFarlane D Nordell B. Cortical activation during rhythmic hand movements performed under three types of control: an fMRI study. Cogn Affect Behav Neurosci. (2002) 2:271–81. 10.3758/CABN.2.3.271 12775191

[54] Buetefisch CM Revill KP Shuster L Hines B Parsons M. Motor demand-dependent activation of ipsilateral motor cortex. J Neurophysiol. (2014) 112:999–1009 10.1152/jn.00110.2014 24848477 PMC4122744

[55] Newton JM Sunderland A Gowland PA. fMRI signal decreases in ipsilateral primary motor cortex during unilateral hand movements are related to duration and side of movement. Neuroimage. (2005) 24:1080–7. 10.1016/j.neuroimage.2004.10.003 15670685

[56] Verstynen T Diedrichsen J Albert N Aparicio P Ivry RB. Ipsilateral motor cortex activity during unimanual hand movements relates to task complexity. J Neurophysiol. (2005) 93:1209–22. 10.1152/jn.00720.2004 15525809

[57] Veverka T Hluštík P Tomášová Z Hok P Otruba P Král M. BoNT-A related changes of cortical activity in patients suffering from severe hand paralysis with arm spasticity following ischemic stroke. J Neurol Sci. (2012) 319:89–95. 10.1016/j.jns.2012.05.008 22687958

[58] Gracies J Elovic E McGuire J Simpson D. Traditional pharmacological treatments for spasticity part I: local treatments. Muscle Nerve Suppl. (1997) 20:61–91. 10.1002/(SICI)1097-4598(1997)6+<61::AID-MUS6>3.0.CO;2-H 9826983

[59] Marciniak CM Harvey RL Gagnon CM Duraski SA Denby FA McCarty S. Does botulinum toxin type A decrease pain and lessen disability in hemiplegic survivors of stroke with shoulder pain and spasticity?: a randomized, double-blind, placebo-controlled trial. Am J Phys Med Rehabil. (2012) 91:1007–19. 10.1097/PHM.0b013e31826ecb02 23064478

[60] Rosales RL Kong KH Goh KJ Kumthornthip W Mok VC Delgado-De Los Santos MM. Botulinum toxin injection for hypertonicity of the upper extremity within 12 weeks after stroke: a randomized controlled trial. Neurorehabil Neural Repair. (2012) 26:812–21. 10.1177/1545968311430824 22371239

[61] Cramer SC Finklestein SP Schaechter JD Bush G Rosen BR. Activation of distinct motor cortex regions during ipsilateral and contralateral finger movements. J Neurophysiol. (1999) 81:383–7. 10.1152/jn.1999.81.1.383 9914297

[62] Du J Hu J Hu J Xu Q Zhang Q Liu L. Aberrances of cortex excitability and connectivity underlying motor deficit in acute stroke. Neural Plast. (2018) 2018:1318093. 10.1155/2018/1318093 30420876 PMC6215555

[63] Nair DG Hutchinson S Fregni F Alexander M Pascual-Leone A Schlaug G. Imaging correlates of motor recovery from cerebral infarction and their physiological significance in well-recovered patients. Neuroimage. (2007) 34:253–63. 10.1016/j.neuroimage.2006.09.010 17070707 PMC2577311

[64] Willer C Ramsay SC Wise RJ Friston KJ Frackwiak RS. Individual patterns of functional reorganization in the human cerebral cortex after capsular infraction. Ann Neurol. (1993) 33:181–9. 10.1002/ana.410330208 8434880

[65] Cauraugh JH Summers JJ. Neural plasticity and bilateral movements: a rehabilitation approach for chronic stroke. Prog Neurobiol. (2005) 75:309–20. 10.1016/j.pneurobio.2005.04.001 15885874

[66] Johansen-Berg H Rushworth MFS Bogdanovic MD Kischka U Wimalaratna S Matthews PM. The role of ipsilateral premotor cortex in hand movement after stroke. Proc Natl Acad Sci USA. (2002) 99:14518–23. 10.1073/pnas.222536799 12376621 PMC137915

[67] Michielsen ME Selles RW van der Geest JN Eckhardt M Yavuzer G Stam HJ. Motor recovery and cortical reorganization after mirror therapy in chronic stroke patients: a phase II randomized controlled trial. Neurorehabil Neural Repair. (2011) 25:223–33. 10.1177/1545968310385127 21051765

[68] Schaechter JD Kraft E Hilliard TS Dijkhuizen RM Benner T Finklestein SP. Motor recovery and cortical reorganization after constraint-induced movement therapy in stroke patients: a preliminary study. Neurorehabil Neural Repair. (2002) 16:326–38. 10.1177/154596830201600403 12462764

[69] Calautti C Baron J. Functional neuroimaging studies of motor recovery after stroke in adults: a review. Stroke. (2003) 34:1553–66. 10.1161/01.STR.0000071761.36075.A6 12738893

[70] Favre I Zeffiro TA Detante O Krainik A Hommel M Jaillard A. Upper limb recovery after stroke is associated with ipsilesional primary motor cortical activity: a meta-analysis. Stroke. (2014) 45:1077–83. 10.1161/STROKEAHA.113.003168 24525953

[71] Dodd KC Nair VA Prabhakaran V. Role of the Contralesional vs. Ipsilesional hemisphere in stroke recovery. Front Hum Neurosci. (2017) 11:469. 10.3389/fnhum.2017.00469 28983244 PMC5613154

[72] Buetefisch CM. Role of the contralesional hemisphere in post-stroke recovery of upper extremity motor function. Front Neurol. (2015) 6:214. 10.3389/fneur.2015.00214 26528236 PMC4607877

[73] Zheng X Sun L Yin D Jia J Zhao Z Jiang Y. The plasticity of intrinsic functional connectivity patterns associated with rehabilitation intervention in chronic stroke patients. Neuroradiology. (2016) 58:417–27. 10.1007/s00234-016-1647-4 26820451

[74] Schulz R Buchholz A Frey BM Bönstrup M Cheng B Thomalla G. Enhanced effective connectivity between primary motor cortex and intraparietal sulcus in well-recovered stroke patients. Stroke. (2016) 47:482–9. 10.1161/STROKEAHA.115.011641 26742802

[75] Fan Y Wu C Liu H Lin K Wai Y Chen Y. Neuroplastic changes in resting-state functional connectivity after stroke rehabilitation. Front Hum Neurosci. (2015) 9:546. 10.3389/fnhum.2015.00546 26557065 PMC4617387

[76] Carter AR Astafiev SV Lang CE Connor LT Rengachary J Strube MJ. Resting interhemispheric functional magnetic resonance imaging connectivity predicts performance after stroke. Ann Neurol. (2010) 67:365–75. 10.1002/ana.21905 20373348 PMC2927671

[77] Pundik S McCabe JP Hrovat K Fredrickson AE Tatsuoka C Feng IJ. Recovery of post stroke proximal arm function, driven by complex neuroplastic bilateral brain activation patterns and predicted by baseline motor dysfunction severity. Front Hum Neurosci. (2015) 9:394. 10.3389/fnhum.2015.00394 26257623 PMC4510426

[78] Ohtsuka H Matsuzawa D Ishii D Shimizu E. Longitudinal follow-up of mirror movements after stroke: a case study. Case Rep Neurol Med. (2015) 2015:354134. 10.1155/2015/354134 26649211 PMC4663000

[79] Nelles G Cramer SC Schaechter JD Kaplan JD Finklestein SP. Quantitative assessment of mirror movements after stroke. Stroke. (1998) 29:1182–7. 10.1161/01.STR.29.6.1182 9626292

[80] Ejaz N Xu J Branscheidt M Hertler B Schambra H Widmer M. Evidence for a subcortical origin of mirror movements after stroke: a longitudinal study. Brain. (2018) 141:837–47. 10.1093/brain/awx384 29394326 PMC5837497

[81] Hiragami S Inoue Y Harada K. Minimal clinically important difference for the Fugl-Meyer assessment of the upper extremity in convalescent stroke patients with moderate to severe hemiparesis. J Phys Ther Sci. (2019) 31:917–21. 10.1589/jpts.31.917 31871377 PMC6879402

[82] Hesse S Reiter F Konrad M Jahnke MT. Botulinum toxin type A and short-term electrical stimulation in the treatment of upper limb flexor spasticity after stroke: a randomized, double-blind, placebo-controlled trial. Clin Rehabil. (1998) 12:381–8. 10.1191/026921598668275996 9796928

[83] Hesse S Brandl-Hesse B Bardeleben A Werner C Funk M. Botulinum toxin A treatment of adult upper and lower limb spasticity. Drugs Aging. (2001) 18:255–62. 10.2165/00002512-200118040-00003 11341473

[84] Hurvitz EA Conti GE Brown SH. Changes in movement characteristics of the spastic upper extremity after botulinum toxin injection. Arch Phys Med Rehabil. (2003) 84:444–54. 10.1053/apmr.2003.50001 12638115

